# Morphological Features in Eyes with Prominent Corneal Endothelial Cell Loss Associated with Primary Angle-Closure Disease

**DOI:** 10.3390/jcm14155364

**Published:** 2025-07-29

**Authors:** Yumi Kusumi, Masashi Yamamoto, Masaki Fukui, Masakazu Yamada

**Affiliations:** Department of Ophthalmology, Kyorin University School of Medicine, Mitaka 181-8611, Japan; kusumi@ks.kyorin-u.ac.jp (Y.K.); myamamoto@ks.kyorin-u.ac.jp (M.Y.); masaki-fukui@ks.kyorin-u.ac.jp (M.F.)

**Keywords:** bullous keratopathy, cornea, endothelium, optical coherence tomography, primary angle-closure disease

## Abstract

**Background:** Patients with primary angle-closure disease (PACD), those with no history of acute angle-closure glaucoma or laser iridotomy, rarely present with prominent corneal endothelial cell density (CECD) loss. To identify factors associated with decreased CECD in PACD, anterior segment parameters were compared in patients with PACD and normal CECD and patients with PACD and decreased CECD, using anterior segment optical coherence tomography (AS-OCT). **Patients and Methods:** Ten patients with PACD and CECD of less than 1500/mm^2^ without a history of cataract surgery, acute angle-closure glaucoma, or prior laser glaucoma procedures were identified at the Kyorin Eye Center from January 2018 to July 2023. Patients with an obvious corneal guttata or apparent corneal edema were also excluded. Seventeen patients with PACD and normal CECD (normal CECD group) were used as the control. Simultaneous biometry of all anterior segment structures, including the cornea, anterior chamber, and iris, were assessed using a swept-source AS-OCT system. **Results:** Corneal curvature radius was significantly larger in the decreased CECD group compared with the corneal refractive power in the normal CECD group (*p* = 0.022, Mann–Whitney test). However, no significant differences were detected in other anterior segment morphology parameters. Multiple regression analysis with CECD as the dependent variable revealed that a large corneal curvature radius was a significant explanatory variable associated with corneal endothelial loss. **Conclusions:** Flattened corneal curvature may be a risk factor for corneal endothelial loss in patients with PACD.

## 1. Introduction

Corneal endothelium plays an important role in preventing edema of the corneal stroma and maintaining the transparency of the cornea [1]. In humans, corneal endothelial cells do not proliferate after birth and decrease with aging. It has been estimated that between the ages of 20 and 80 years the reduction in cell density averages 0.52% per year [2]. In addition to aging, it is known that CECD is also affected by gender and race. The lack of replicative potential has immense consequences in clinical settings such as Fuchs endothelial corneal dystrophy, trauma, ocular surgery, and several ocular conditions [2,3].

Primary angle-closure disease (PACD) is one of the conditions that may affect decrease in corneal endothelial cells [3,4,5]. PACD is clinically classified into subgroups, including primary angle-closure suspect, primary angle closure, and primary angle-closure glaucoma [6,7,8]. Of these, the association between decreased CECD in patients with PACD and a history of acute angle-closure glaucoma and laser iridotomy (LI) is well known [9,10,11,12,13]. Sihota et al. reported a mean corneal endothelial cell loss of 35.1% in eyes with a history of acute angle-closure glaucoma [10]. Li and associates reported that 12.3% of eyes with a history of acute attacks had severe corneal endothelial damage [11]. Laser iridotomy (LI) may also be associated with corneal endothelial loss [12,13,14]. Post-LI bullous keratopathy is common in China and Japan [13]. According to a nationwide survey on corneal transplantation in Japan, LI was the second most common cause of bullous keratopathy requiring keratoplasty [14]. However, opinions in Western countries regarding prominent corneal endothelial loss due to LI have been skeptical [15,16]. Recent clinical studies tracking the degree of corneal endothelial cell loss over time after LI reveal that the decrease in CECD is limited [17,18].

The effect of PACD with no history of acute angle-closure glaucoma or LI on CECD has been investigated [19,20,21,22]. Most reports acknowledge that the impact of PACD on the corneal endothelium is minimal [19,20,21]. However, Sakai et al. reported rare cases of angle-closure glaucoma with prominent decrease in CECDs [22]. We have so far encountered several cases of PACD without a history of acute angle-closure glaucoma or LI, but accompanied by decreased CECD. We hypothesized that these rare cases of PACD accompanied by a prominent decrease in CECD had certain morphological characteristics. In the present study, we compared the anterior segment parameters of PACD in eyes with normal CECD and eyes with decreased CECD using anterior segment optical coherence tomography (AS-OCT).

## 2. Materials and Methods

The medical records of patients who were diagnosed with PACD at the Kyorin Eye Center from January 2018 to July 2023 were reviewed. The inclusion criteria for this study were patients aged 50 years or older, intraocular pressure of <21 mmHg, peripheral anterior chamber depth of grade 2 or less by Van Herick’s technique, and no history of prior glaucoma medication or intervention, with CECD of less than 1500 cells/mm^2^. The definition of decreased CECD was based on the grading for corneal endothelial damage by Kinoshita and associates [23]. The exclusion criteria were as follows: a history of cataract surgery, acute attack of angle-closure glaucoma, prior laser glaucoma procedures including LI, presence of obvious corneal guttata, or clinically apparent corneal edema. Seventeen eyes from 10 patients were identified as the decreased CECD group, whereas three eyes were excluded because they were pseudophakic at the initial visit. Seventeen eyes from 17 PACD patients with CECD of 2000/mm^2^ or higher (normal CECD group) during the same period were selected as a control [23].

The results of ophthalmic examinations, including visual acuity testing, slit lamp examination, intraocular pressure measurements, Goldmann applanation tonometry, anterior segment optical coherence tomography (AS-OCT), and specular microscopy, were collected from the medical records. A noncontact specular microscope (CellChek 20-1, Konan Medical, Nishinomiya, Japan) was used to examine the corneal endothelium. This device performs an automated analysis of cell parameters, including the CECD, using the center method.

The CASIA2 swept-source AS-OCT system (Tomey Corp., Nagoya, Japan) was used for the AS-OCT examination. This AS-OCT device has a swept-source laser that operates at a central wavelength of 1310 nm and a scan rate of 50,000 A-scans per second. The maximum imaging area of the AS-OCT device is 16.0 mm × 16.0 mm, and the maximum imaging depth is 11.0 mm. The simultaneous biometry of all anterior segment structures, including the cornea, anterior chamber, and iris, can be performed using the AS-OCT device. The AS-OCT images were analyzed using the SSOCT viewer (version 9.0, Tomey, Nagoya, Japan). Scleral spurs were identified by a prominent inner extension of the sclera and the iris recess apex was marked manually. The following anterior segment parameters were automatically analyzed by the algorithm: anterior chamber depth (ACD), anterior chamber volume, anterior chamber width, angle opening distance at 750 μm from the scleral spur (AOD750), and trabecular iris angle at 750 μm (TIA750), which was measured with the apex in the iris recess with the arms of the angle passing through a point on the trabecular 750 μm meshwork from the scleral spur and the perpendicular point on the iris [23].

Statistical analysis was performed using Prism 9 Version 9.5.0 (GraphPad Software, Boston, MA, USA). The decreased CECD and normal CECD groups were compared using the Mann–Whitney test, mixed-effect model or Fischer’s exact test. The variables related to CECD as a dependent variable were identified using multiple linear regression analysis. Statistical significance was set at *p* < 0.05.

## 3. Results

Ten patients with PACD accompanied by decreased CECD, but without a history of acute glaucoma attack or LI (decreased CECD group), were identified from the review of medical records. Of these, three eyes from three patients were excluded for further analysis because they were pseudophakic at the initial visit. Seventeen eyes from 17 PACD patients with CECD of 2000 cells/mm^2^ or higher (normal CECD group) during the same period were selected as a control. Table 1 shows the patient demographics. No differences in age, gender, or intraocular pressure were detected between the two groups. CECD decreased significantly in the decreased group (702.6 ± 209.8 cells/mm^2^) compared to the normal group (2609.0 ± 307.6 cells/mm^2^), but central corneal thicknesses were similar in the two groups.

The anterior segment parameters determined by AS-OCT in the decreased CECD and normal CECD groups are shown in Table 2. Corneal curvature radius was significantly larger in the decreased CECD group compared with the normal CECD group (*p* = 0.029, mixed-effect model). There were, however, no significant differences in ACD, ACW, AOD750, and TIA750.

To determine the factors related to CECD, multiple regression analysis with CECD as the dependent variable was performed. As shown in Table 3, sex and corneal curvature radius were significant explanatory variables associated with CECD loss.

## 4. Discussion

PACD may have a negative effect on CECD [3,4,5]. It is well recognized that a history of acute attack of angle-closure glaucoma and LI is associated with corneal endothelial loss in PACD [10,11,12,13,14]. However, the association between PACD and decreased CECD in those with no history of acute angle-closure glaucoma or LI is controversial [19,20,21,22]. Most reports acknowledge that the impacts of PACD on the corneal endothelium are few, if there are any. However, there exist rare cases of PACD with decreased CECD despite no history of acute glaucoma attacks or LI [22]. The objective of the present study was to identify morphological factors other than acute angle-closure glaucoma and LI that are related to decreased CECD in patients with PACD.

Corneal curvature radius was significantly larger in the decreased CECD group compared with the normal CECD group. Moreover, the multivariate analysis with CECD as the dependent variable also revealed that corneal curvature radius was a significant explanatory variable associated with CECD loss. Although the number of cases was limited, both analyses suggest that a flattened corneal shape is associated with endothelial cell loss in PACD. Cases with obvious corneal edema were not included in this study. Therefore, the differences in corneal curvature radius cannot be attributed to morphological changes secondly caused by corneal edema. Other anterior segment morphologic parameters, including ACD, ACW, AOD750, and TIA750, were not associated with CECD loss. Thus, the morphology of the central cornea, rather than the angle or the peripheral cornea morphology, may be associated with corneal endothelial loss. The exact mechanism linking a flat cornea to corneal endothelial damage is unclear. In eyes with a flattened corneal shape, intermittent contact between the endothelium and the anterior surface of the lens may be more likely to occur. Alternatively, a flat corneal shape increases the contact area between the corneal endothelium and the iris or anterior surface of the lens. The detailed mechanism remains to be investigated.

In the multivariate analysis with CECD as the dependent variable, gender (female) was a significant explanatory variable associated with CECD loss. However, this study had a small sample size, with most participants (both cases and controls) being female. Therefore, the significance of this finding needs further investigation with a larger sample size. If gender is related to CECD loss in PACD, this may be due to the fact that PACD is more prevalent in older women [24,25]. Another possibility is that female hormones affect the corneal endothelium. Several clinical and experimental studies have reported that estrogen is one of the causes of Fuchs endothelial corneal dystrophy being more prevalent in women [26,27,28,29]. The issue of gender differences is an interesting topic that warrants further research.

There are several limitations in this study. First, the number of cases in this study is limited, making it difficult to draw definitive conclusions. This is due to the rarity of the cases involved. Only a few cases of decreased CECD were detected among the PACD cases, and cases without a history of acute angle-closure glaucoma attacks or LI are rare. Second, the cross-sectional design of the study did not allow for the assessment of changes over time. Patients with PACD, normal intraocular pressure, and no symptoms may not visit ophthalmology clinics. Additionally, patients with PACD may require surgical interventions such as cataract surgery or LI. Thus, longitudinal changes over a long period of time cannot be observed in these patients. Another possible limitation is possible past elevations of intraocular pressure or spontaneously resolved acute attacks of glaucoma that were not reported. Although the medical interview checks for any history of angle-closure glaucoma attacks, a history of slight, asymptomatic elevation in intraocular pressure may be overlooked. However, it is difficult to completely exclude such a possibility in clinical studies.

Although this study’s small sample size necessitates further research, we believe that our findings are clinically significant because they highlight the presence of PACD cases exhibiting reduced CECD without prior acute glaucoma attacks or LI. In addition, this study suggests that flattened corneal shape is related to corneal endothelial loss in PACD. The mechanisms and risk factors for corneal endothelial loss in PACD should be investigated in the future through analyses of larger numbers of cases in further studies.

## 5. Conclusions

It is known that a history of acute glaucoma attack and LI is a factor that can cause corneal endothelial cell loss in PACD. However, there are cases of PACD that exhibit decreased CECD without these factors. In this study, we have shown that flattened corneal curvature may be a risk factor for corneal endothelial loss in patients with PACD.

## Figures and Tables

**Table 1:** Patient demographics.

Groups	Decreased CECD Group	Normal CECD Group	*p* Value
Age (years)	76.8 ± 8.4 (57–89)	73.4 ± 8.9 (60–89)	0.190 *
Sex (female:male)	10:0	14:3	0.271 **
IOP (mmHg)	13.3 ± 2.2 (10–18)	14.5 ± 2.5 (9–18)	0.153 ***
CCT (µm)	538.6 ± 49.6 (478–609)	536.6 ± 31.8 (493–602)	0.849 ***
CECD (cells/mm^2^)	702.6 ± 209.8 (390–1285)	2609.0 ± 307.6 (2038–3185)	<0.0001 ***

Age, IOP, CCT, and CECD are presented as average ± SD (range). * Mann–Whitney test. ** Fisher’s exact test. *** Mixed-effect model. CECD: corneal endothelial cell density, IOP: intraocular pressure, CCT: central corneal thickness.

**Table 2:** Anterior segment parameters in the decreased CECD and normal CECD groups.

Variables	Decreased CECD Group	Normal CECD Group	*p* Value *
Corneal curvature radius (mm)	7.70 ± 0.25	7.57 ± 0.16	0.029
ACD (mm)	2.00 ± 0.15	2.00 ± 0.20	0.987
ACW (mm)	10.84 ± 0.49	10.92 ± 0.55	0.345
AOD750 (mm)	0.26 ± 0.09	0.19 ± 0.097	0.128
TIA750 (°)	12.11 ± 3.35	9.78 ± 3.71	0.393

All parameters are represented as average ± SD (range). * Mixed-effect model. CECD: corneal endothelial cell density, ACD: anterior chamber depth, ACW: anterior chamber width, AOD750: angle opening distance 750 μm from the scleral spur, TIA750: trabecular iris angle 750 μm from the scleral spur.

**Table 3:** Multiple regression analysis with corneal endothelial cell density as the dependent variable.

Variables	Coefficient	95% Confidence Interval	*p* Value
Age	−45.52	−94.05, 2.131	0.060
Sex (male)	1424	173.9, 2680	0.027
IOP (mmHg)	111.7	−37.35, 258.8	0.129
CCT (μm)	5.671	−4.757, 16.10	0.270
Corneal curvature radius (mm)	−2816	−5211, −420.8	0.024
ACD (mm)	−196.5	−5362, 4969	0.938
ACW (mm)	−1123	−2533, 285.8	0.112
AOD750 (mm)	3024	−38,673, 44,722	0.881
TIA750 (°)	−377.2	−1144, 389.5	0.317

IOP: intraocular pressure, CCT: central corneal thickness, ACD: anterior chamber depth, ACW: anterior chamber width, AOD750: angle opening distance 750 μm from the scleral spur, TIA750: trabecular iris angle 750 μm from the scleral spur.

## Data Availability

The original contributions presented in the study are included in the article; further inquiries can be directed to the corresponding author.

## Abbreviations

The following abbreviations are used in this manuscript:

PACD	Primary angle-closure disease
CECD	Corneal endothelial cell density
AS-OCT	Anterior segment optical coherence tomography
LI	Laser iridotomy
ACD	Anterior chamber depth
AOD750	Angle opening distance at 750 μm from the scleral spur
TIA750	Trabecular iris angle at 750 μm
IOP	Intraocular pressure
CCT	Corneal central thickness
